# Supplementary material for: Structural insights into the ubiquitylation strategy of the oligomeric CRL2FEM1B E3 ubiquitin ligase
Source: EMBO J. 2024 Feb 15;43(6):1089–109. doi: 10.1038/s44318-024-00047-y (PMC10943247; doi:10.1038/s44318-024-00047-y)
Supplement: Supplementary file 1 — Appendix [file 44318_2024_47_MOESM1_ESM.pdf]

Appendix for

Structural Insights into the Ubiquitylation Strategy of the Oligomeric CRL2^{FEM1B} E3 Ubiquitin Ligase

Zonglin Dai[1,2,†], Ling Liang[1,3,†], Weize Wang[1,4,†], Peng Zuo[1,2], Shang Yu[3], Yaqi Liu[5], Xuyang Zhao[1], Yishuo Lu[1,4], Yan Jin[1], Fangting Zhang[6], Dian Ding[1], Weiwei Deng[7] and Yuxin Yin[1,2,4,6*]

*Correspondence: Yuxin Yin.
Email: yinyuxin@hsc.pku.edu.cn.

Table of Contents:

Appendix References Page 2
Appendix Figure S1 to S11 Page 3-14
Appendix Table S1 Page 15

Appendix References

Glaser F, Pupko T, Paz I, Bell RE, Bechor-Shental D, Martz E, Ben-Tal N (2003) ConSurf: identification of functional regions in proteins by surface-mapping of phylogenetic information. *Bioinformatics* 19: 163-164

Mayrose I, Graur D, Ben-Tal N, Pupko T (2004) Comparison of site-specific rate-inference methods for protein sequences: empirical Bayesian methods are superior. *Mol Biol Evol* 21: 1781-1791

Pupko T, Bell RE, Mayrose I, Glaser F, Ben-Tal N (2002) Rate4Site: an algorithmic tool for the identification of functional regions in proteins by surface mapping of evolutionary determinants within their homologues. *Bioinformatics* 18 Suppl 1: S71-77

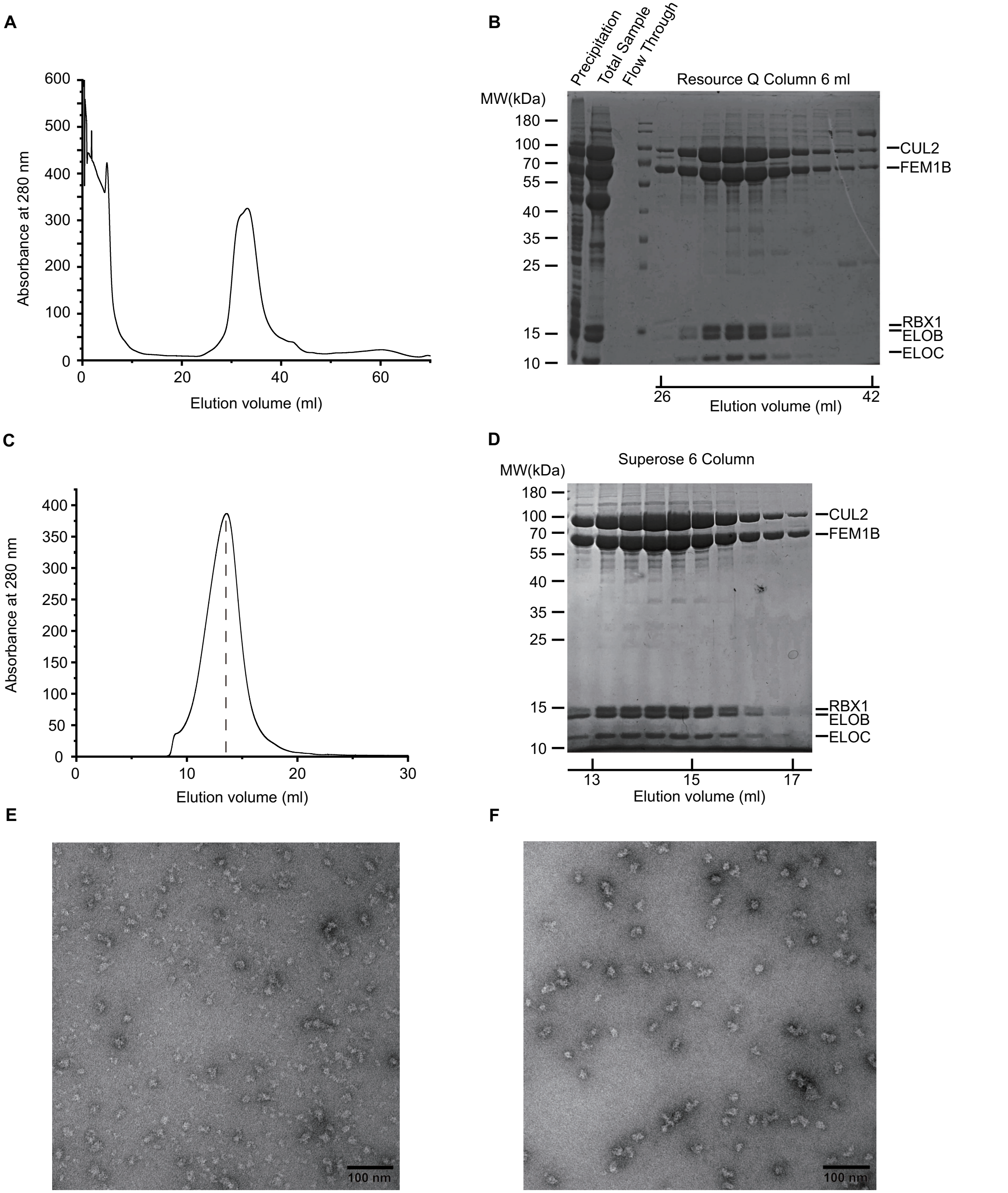

Appendix Figure S1. Protein purification of CRL2^{FEM1B} E3 ubiquitin ligase.

(***A***) Anion-exchange chromatography of CRL2^{FEM1B}.

(***B***) The SDS-PAGE gel filtration outcomes were obtained by analyzing aliquots derived from samples subjected to anion-exchange chromatography.

(***C***) Size exclusion chromatography of CRL2^{FEM1B}.

(***D***) Aliquots of CRL2^{FEM1B} taken from size exclusion chromatography analyzed by SDS-PAGE gel filtration.

(***E***) A representative negative-stain micrograph of the CRL2^{FEM1B} complex.

(***F***) A representative negative-stain micrograph of the CRL2^{FEM1B} complex after GraFix.

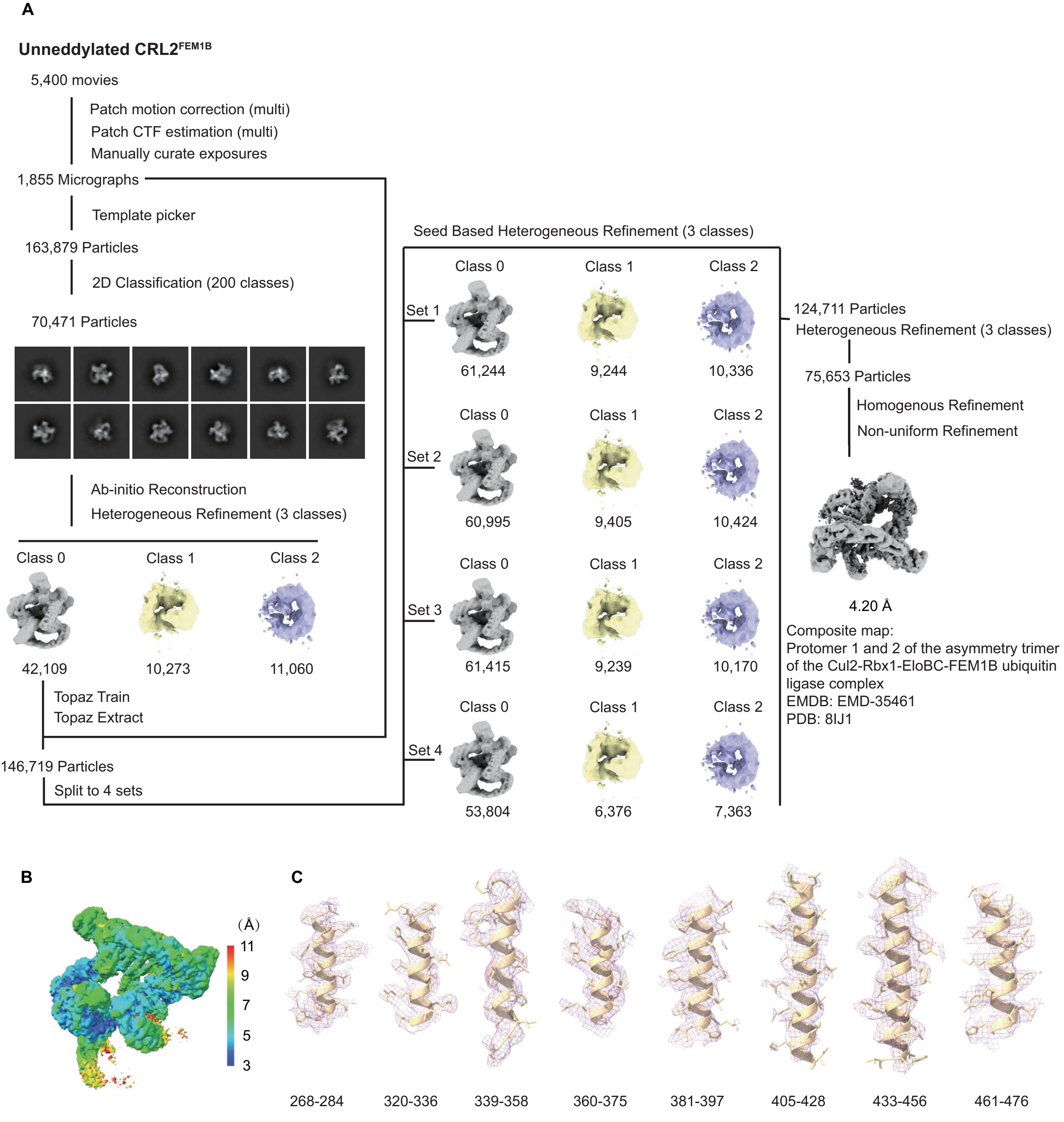

Appendix Figure S2. Structure determination workflow for unneddylated CRL2^{FEM1B}.

(***A***) Workflow for the structure determination with the final cryo-EM maps.

(***B***) 4.2Å cryo-EM map colored according to local resolution.

(***C***) Representative cryo-EM densities of C-terminus domain of FEM1B from 4.2Å cryo-EM map are shown as mesh, with corresponding stick models superimposed.

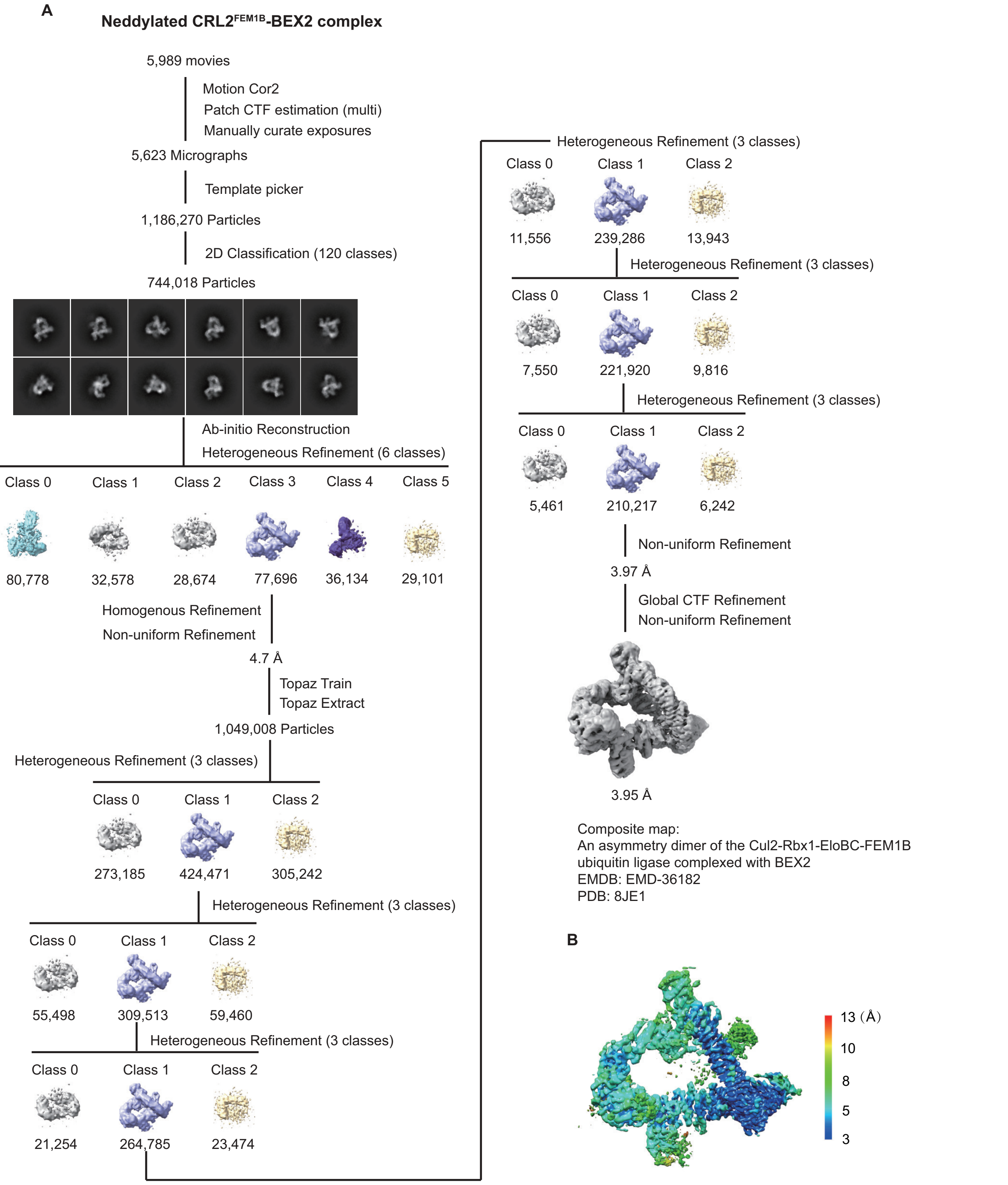

Appendix Figure S3. Structure determination workflow for N8-CRL2FEM1B-BEX2 complex.

(***A***) Workflow for the structure determination with the final cryo-EM map.

(***B***) Cryo-EM map colored according to local resolution.

A

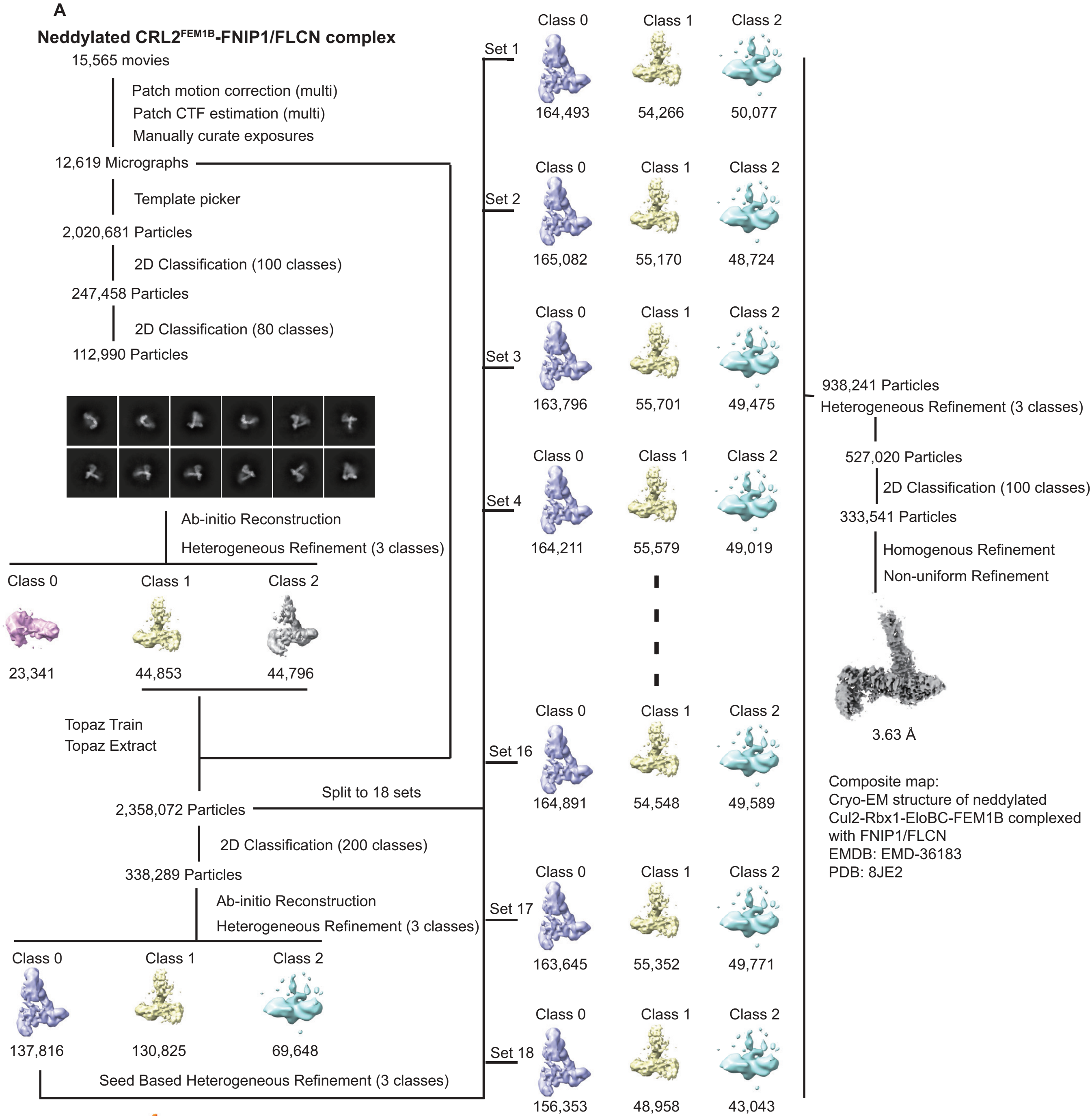

B

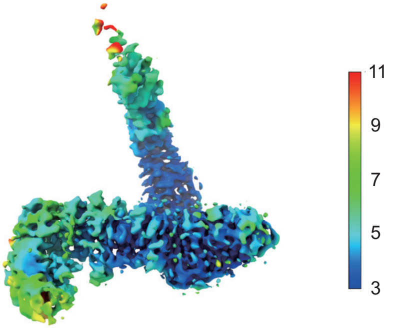

Appendix Figure S4. Structure determination workflow for N8-CRL2FEM1B-FNIP1/FLCN complex.

(***A***) Workflow for the structure determination with the final cryo-EM map.

(***B***) Cryo-EM map colored according to local resolution.

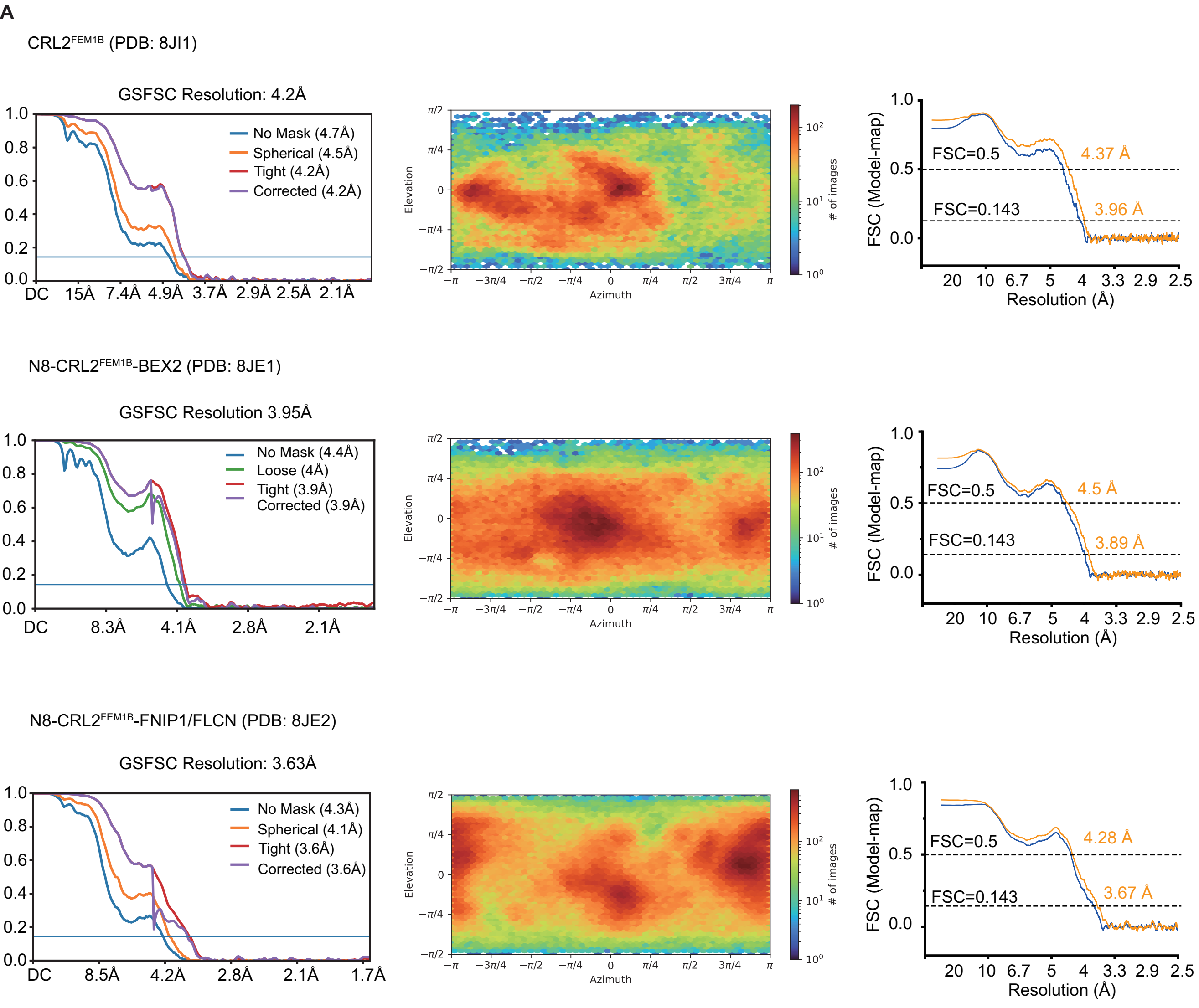

Appendix Figure S5. FSC curves.

(***A***) GSFSCs (left) and viewing angle distributions (middle) of the non-uniform refinement performed in cryoSPARC, and FSC model-map performed in PHENIX (right) are shown.

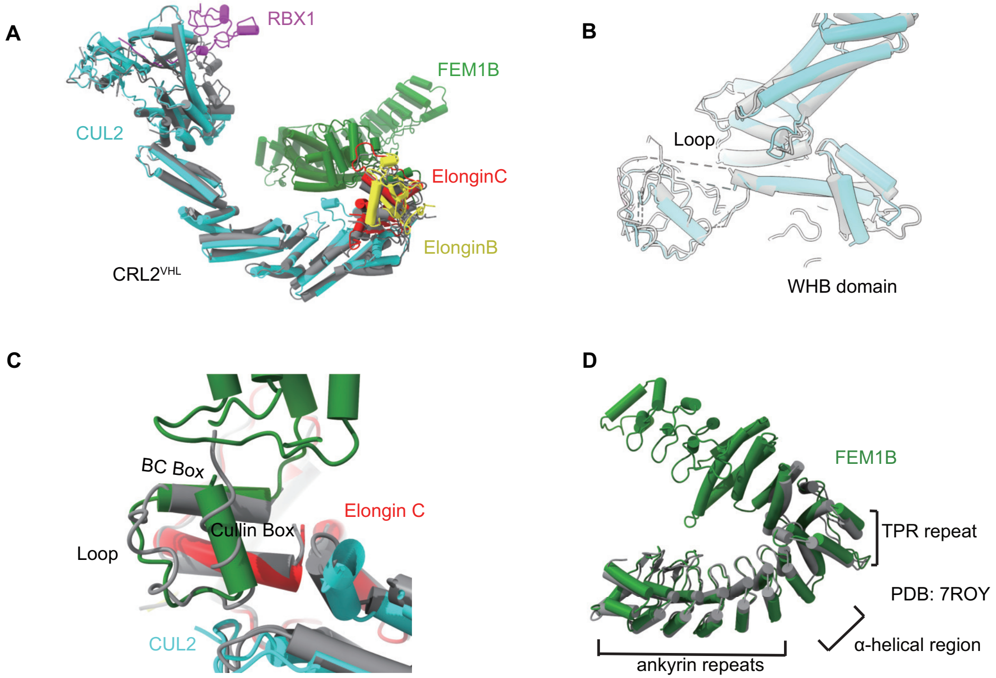

Appendix Figure S6. Architectural organization of CRL2^{FEM1B}.

(***A***) Protomer 1 of CRL2^{FEM1B} has a conformation similar to that of CRL2^{VHL} as shown by the superimposition of the two atomic structures. The five subunits of CRL2^{FEM1B} are each assigned their own color as indicated while CRL2^{VHL} is colored grey.

(***B***) In superimposed structures shown in (A), the loops connecting the WHB domain to the a/b domain of CUL2 are not visible because of flexibility. Models are colored the same as in (A).

(***C***) Superimposed structures show that FEM1B has a canonical VHL box which is subdivided into a BC box (residues 597-608) and a cullin box (residues 618-627) that interact with ELOC and CUL2, respectively. Structures are colored the same as in (A).

(***D***) Atomic structure of FEM1B superimposed on the published crystal structure of the N-terminal domain of FEM1B (PDB: 7ROY).

A

```
FEM1B_HUMAN    1 MEGLAGYVYKAASEGKVLTLAALLLNRSESDIRYLLGYVSQQGGQRSTPLIIAARNGHAKVVRLLLEHYRVQTQQTGTVRFDGYVIDGATALWCAAGAGHFEVVKLLVSHGANVNHTTVT 120
FEM1A_HUMAN    1 -MDLRTAVYNAARDGKLQLLQKLLSGRSREELDELTGEVA----GGGTPLLIAARYGHLDVVEYLVDRCGASVEAGGSVHFDGETIEGAPPLWAASAAGHLDVVRSLLRRGASVNRTTRT 115
FEM1C_HUMAN    1 -MDLKTAVFNAARDGKLRLLTKLLASKSKEEVSSLISEKT----NGATPLLMAARYGHLDMVEFLLEQCSASIEVGGSVNFDGETIEGAPPLWAASAAGHLKVVQSLLNHGASVNNTTLT 115
                   .*   *::** :**:  *  ** .:*..::  * .  :      .***::*** ** .:*. *:::  .. :  *:*.*** .*:**  **.*:.***:.**: *: :**.**.** *

FEM1B_HUMAN  121 NSTPLRAACFDGRLDIVKYLV-ENNANISIANKYDNTCLMIAAYKGHTDVVRYLLEQRADPNAKAHCGATALHFAAEAGHIDIVKELIKWRAAIVVNGHGMTPLKVAAESCKADVVELLL 239
FEM1A_HUMAN  116 NSTPLRAACFDGHLEVVRYLVGEHQADLEVANRHGHTCLMISCYKGHREIARYLLEQGAQVNRRSAKGNTALHDCAESGSLEILQLLLGCKARMERDGYGMTPLLAASVTGHTNIVEYLI 235
FEM1C_HUMAN  116 NSTPLRAACFDGHLEIVKYLV-EHKADLEVSNRHGHTCLMISCYKGHKEIAQYLLEKGADVNRKSVKGNTALHDCAESGSLDIMKMLLMYCAKMEKDGYGMTPLLSASVTGHTNIVDFLT 234
                 ************:*::*:*** *::*::.::*::.:*****:.**** ::.:****: *: * ::  * **** .**:* ::*:: *:   * :  :*:*****  *: : ::::*: *

FEM1B_HUMAN  240 SHAD----------------------------------------------------CDRRSRIEALELLGASFANDRENYDIIKTYHYLYLAMLERFQDGDNILEKEVLPP-IHAYGNRT 306
FEM1A_HUMAN  236 QEQPGQEQVAGGEAQPGLPQEDPSTSQGCAQPQGAPCCSSSPEEPLNGESYESCCPTSREAAVEALELLGATYVDKKR--DLLGALKHWRRAMELRHQG-GEYLPKPEPPQLVLAYDYSR 352
FEM1C_HUMAN  235 HHAQ----------------------------------------------------TSKTERINALELLGATFVDKKR--DLLGALKYWKKAMNMRYSDRTNIISKPVPQTLIMAYDYAK 300
                   .                                                   .:  ::*******::.:.:.  *:: : ::   **  *...  : : *      : **.
                                                                                   Contact domain

FEM1B_HUMAN  307 ECRNPQELESIRQDRDALHMEGLIVRERILGADNIDVSHPIIYRGAVYADNMEFEQCIKLWLHALHLRQKGNR----NTHKDLLRFAQVFSQMIHLN-------ETVKAPDIECVLRCSV 415
FEM1A_HUMAN  353 EVNTTEELEALITDPDEMRMQALLIRERILGPSHPDTSYYIRYRGAVYADSGNFERCIRLWKYALDMQQSNLEPLSPMTASSFLSFAELFSYVLQDRAAKGSLGTQIGFADLMGVLTKGV 472
FEM1C_HUMAN  301 EVNSAEELEGLIADPDEMRMQALLIRERILGPSHPDTSYYIRYRGAVYADSGNFKRCINLWKYALDMQQSNLDPLSPMTASSLLSFAELFSFMLQDR-AKGLLGTTVTFDDLMGILCKSV 419
                 * .. :***.:  * * ::*:.*::****** .: *.*: * ********. :*::**.** :**.::*..       * ..:* **::** ::: .          :   *:  :*  .*

FEM1B_HUMAN  416 LEIEQSMNRVKNISDADVHNAMDNYECNLYTFLYLVCISTKTQCSEEDQCKINKQIYNLIHLDPRTREGFTLLHLAVNSNTPVDDFHTNDVCSFPNALVTKLLLDCGAEVNAVDNEGNSA 535
FEM1A_HUMAN  473 REVERALQLPREPGDS------AQFTKALAIILHLLYLLEKVECTPSQEHLKHQTVYRLLKCAPRGKNGFTPLHMAVDKDTTN--VGRYPVGRFPSLHVVKVLLDCGADPDSRDFDNNTP 584
FEM1C_HUMAN  420 LEIERAIKQTQCPADP------LQLNKALSIILHLICLLEKVPCTLEQDHFKKQTIYRFLKLHPRGKNNFSPLHLAVDKNTTC--VGRYPVCKFPSLQVTAILIECGADVNVRDSDDNSP 531
                  *:*::::  :  .*        :    *  :*:*: :  *. *: .::   :: :*.:::  ** ::.*: **:**:.:*     .    *  **.  *. :*::***: :  * :.*:

FEM1B_HUMAN  536 LHIIVQYNRPISDFLTLHSIIISLVEAGAHTDMTNKQNKTPLDKSTT-GVSEILLKTQMKMSLKCLAARAVRANDINYQDQIPRTLEEFVGFH- 627
FEM1A_HUMAN  585 LHIAAQNNCPA--------IMNALIEAGAHMDATNAFKKTAYELLDEKLLARGTMQPFNYVTLQCLAARALDKNKIPYKGFIPEDLEAFIELH- 669
FEM1C_HUMAN  532 LHIAALNNHPD--------IMNLLIKSGAHFDATNLHKQTASDLLDEKEIAKNLIQPINHTTLQCLAARVIVNHRIYYKGHIPEKLETFVSLHR 617
                 *** .  * *        *:  *:::*** * **  ::*  :      ::.  ::     :*:*****.:  : * *:. **. ** *: :*
                                Contact domain
```

B

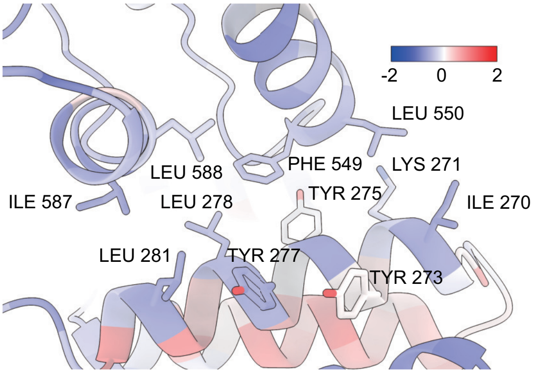

Appendix Figure S7. Conservation within the FEM1 family and evolutionary conservation of FEM1B.

(***A***) Sequence alignment of FEM1B, FEM1A and FEM1C by CLUSTALW. Highly conserved residues are shown with a red background. Residues involved in the trimerization of CRL2^{FEM1B} are underlined and denoted as 'contact domain'. The overall sequence identity is over 30 % among those 3 proteins.

(***B***) Residues involved in trimerization of CRL2^{FEM1B} are not conserved as shown by the atomic structure of FEM1B colored according to evolutionary conservation (Glaser et al, 2003; Mayrose et al, 2004; Pupko et al, 2002). Highly conserved residues are colored in red while residues with low conservation are colored in blue.

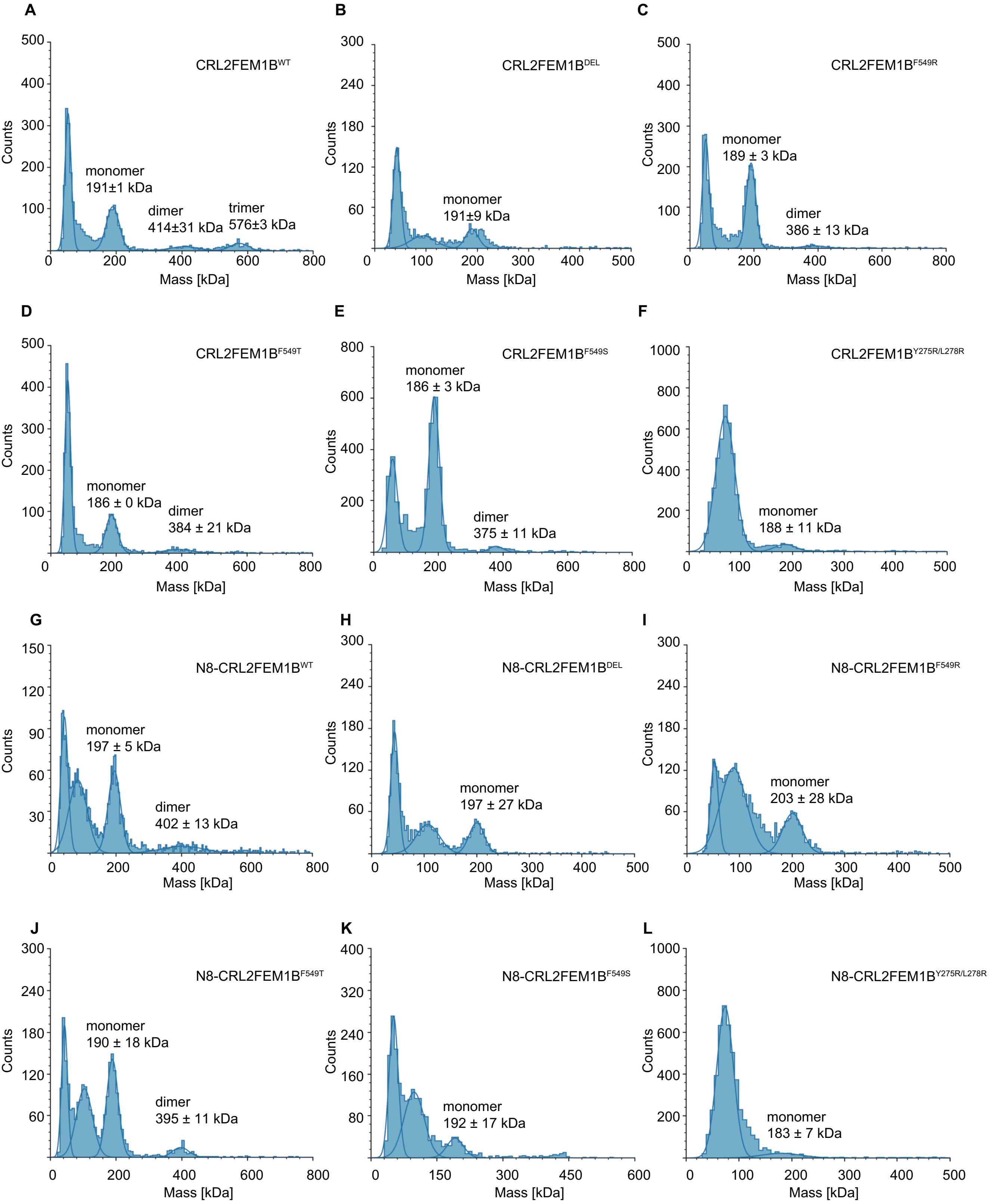

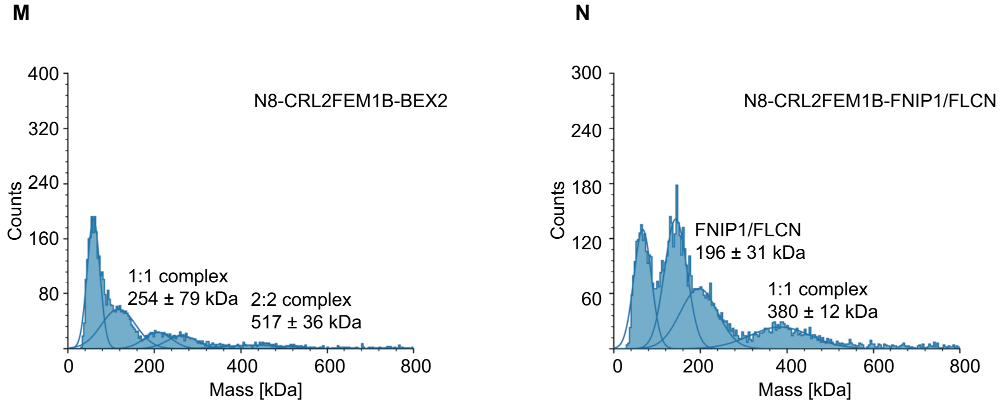

Appendix Figure S8. Molecular masses measured by mass photometry experiments. (n=3)

(***A***) Mass photometry experiment result of wild-type $\text{CRL2}^{\text{FEM1B}}$.

(***B-F***) Molecular weights of oligomerization deficient mutants of $\text{CRL2}^{\text{FEM1B}}$ measured by mass photometry experiments.

(***G***) Molecular mass of N8-$\text{CRL2}^{\text{FEM1B}}$ measured by mass photometry experiment.

(***H-L***) Molecular mass of oligomerization deficient mutants of N8-$\text{CRL2}^{\text{FEM1B}}$ measured by mass photometry experiments.

(***M***) Mass photometry experiment result of N8-$\text{CRL2}^{\text{FEM1B}}$-BEX2 sample in which BEX2 is excessive.

(***N***) Mass photometry experiment result of N8-$\text{CRL2}^{\text{FEM1B}}$-FNIP1/FLCN sample in which FNIP1/FLCN is excessive.

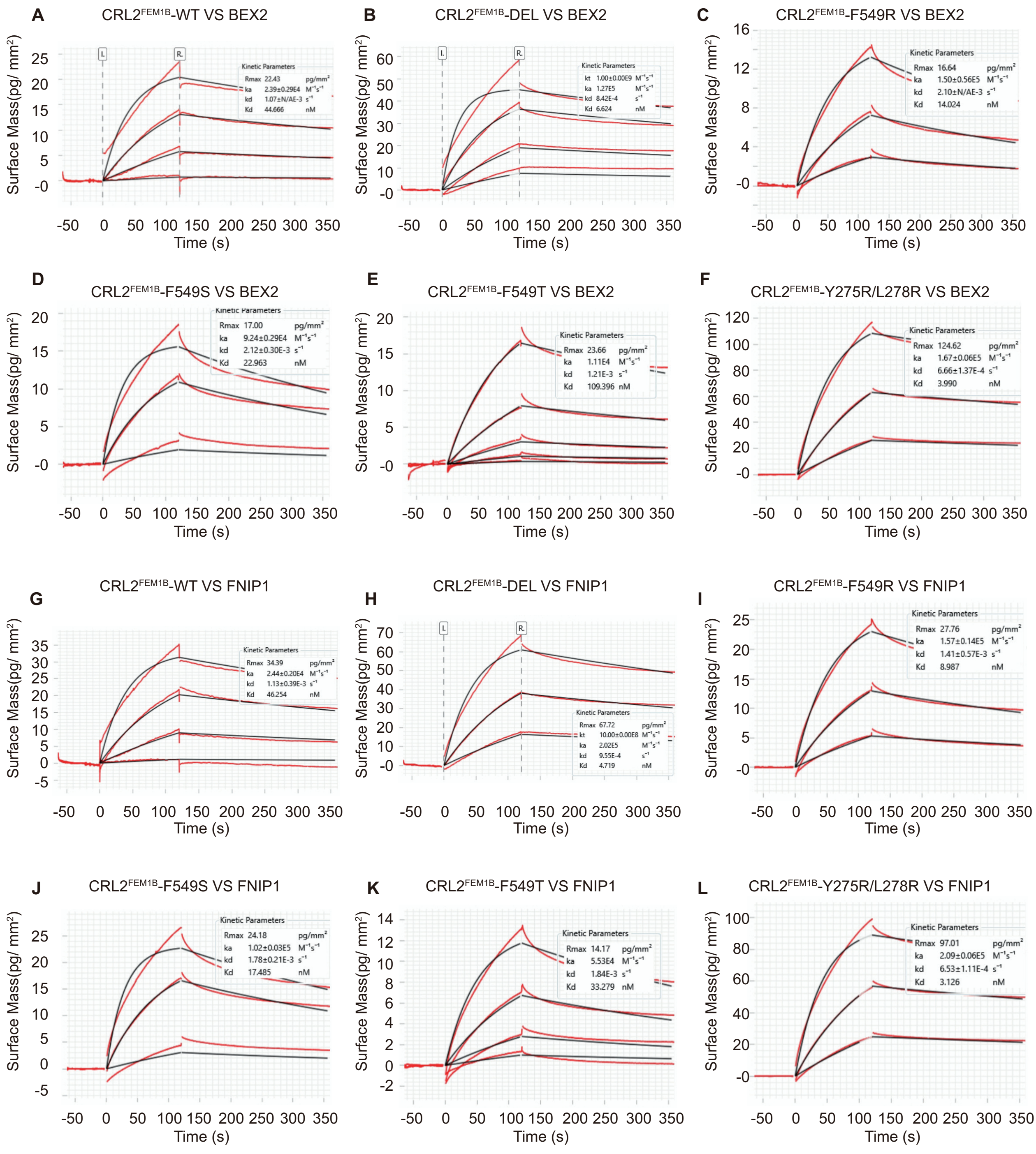

Appendix Figure S9. Substrate binding affinities measured by grating-coupled interferometry experiments.

(***A-F***) CRL2^{FEM1B} and mutants have similar binding affinities to MBP-BEX2, as shown by grating-coupled interferometry experiments.

(***G-L***) CRL2^{FEM1B} and mutants have similar binding affinities to FNIP1/FLCN, as shown by grating-coupled interferometry experiments.

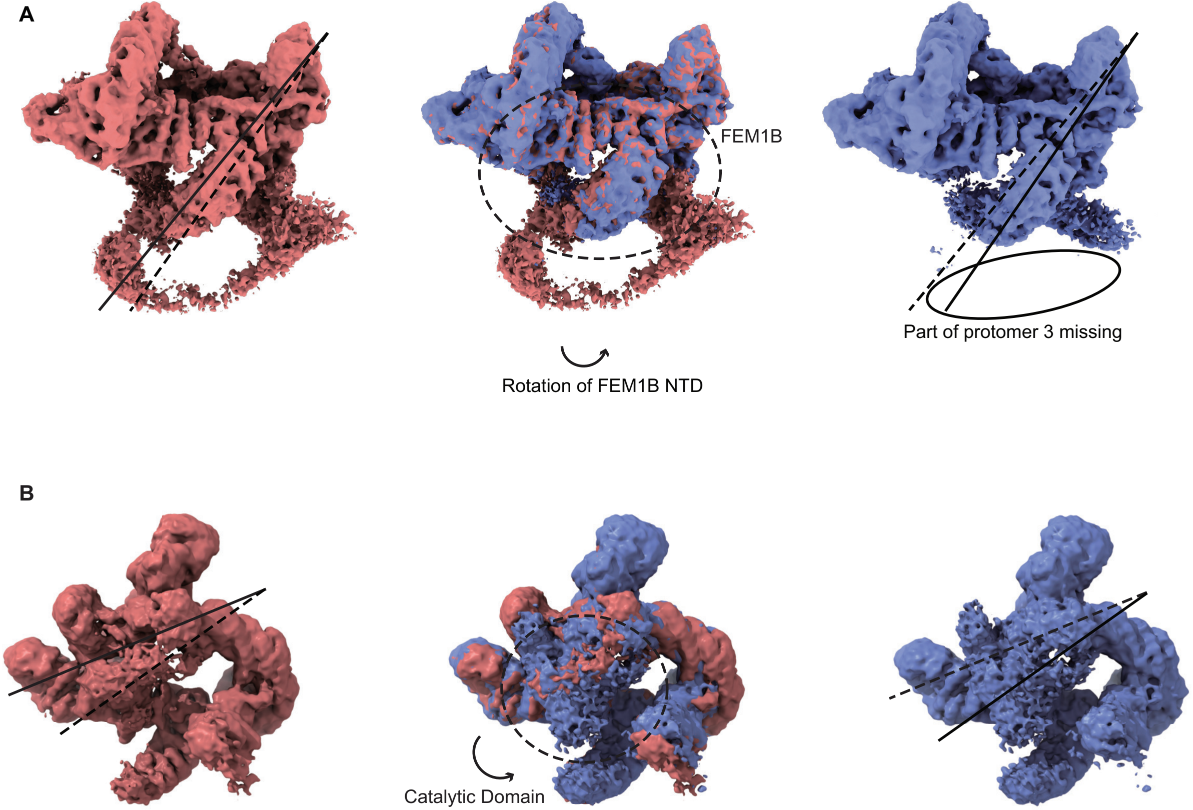

Appendix Figure S10. 3D variability analysis of CRL2^{FEM1B} and N8-CRL2^{FEM1B}-BEX2 complexes.

(***A***) 3DVA analysis of trimeric CRL2^{FEM1B} cryo-EM data by cryoSPARC shows the flexibility of the substrate binding pocket of FEM1B. Broken circled region correspond to the density of FEM1B within trimeric CRL2^{FEM1B} complex.

(***B***) 3DVA analysis of N8-CRL2^{FEM1B}-BEX2 cryo-EM data by cryoSPARC shows the flexibility of the catalytic domain of protomer 2.

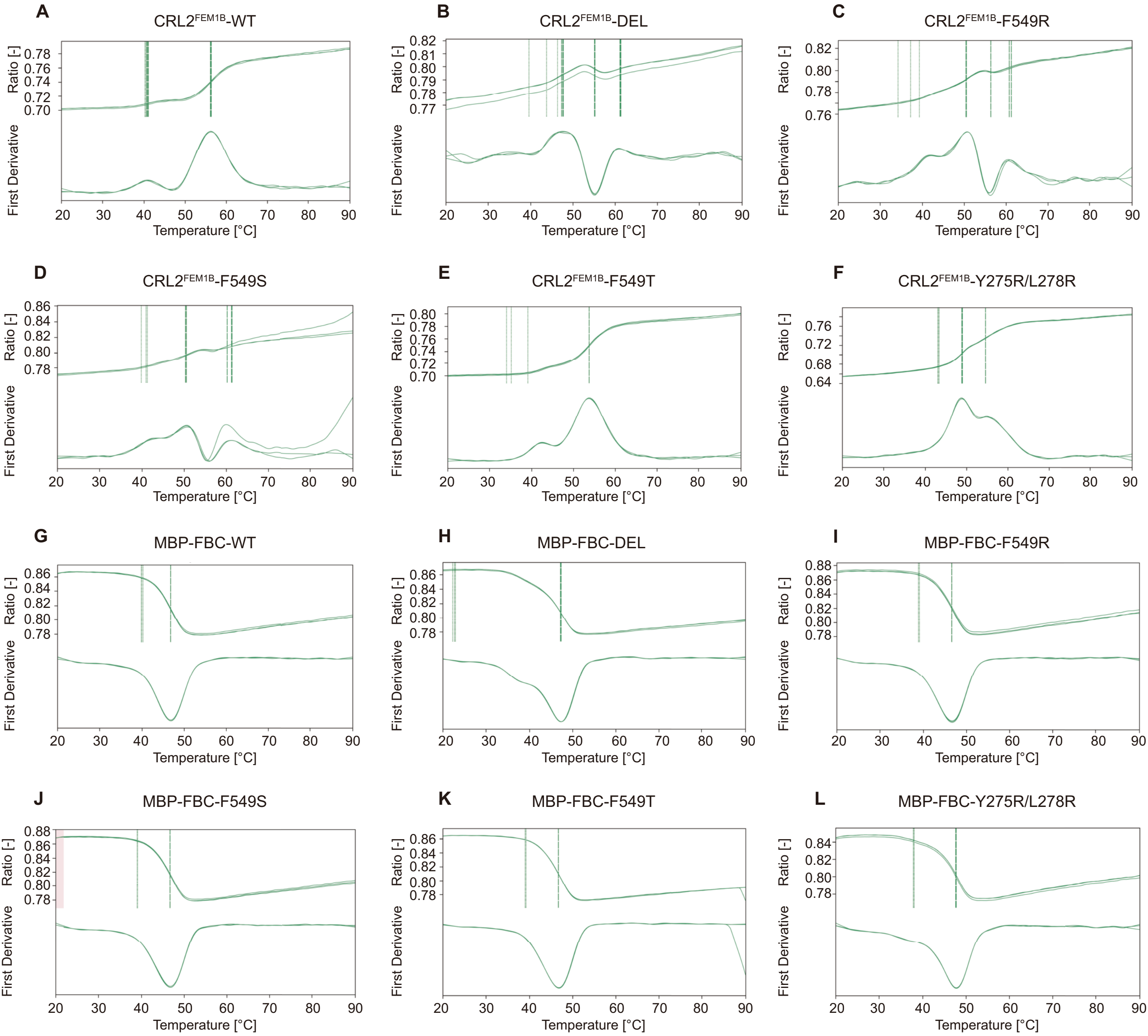

Appendix Figure S11. Nano Differential Scanning Fluorimetry-Based Thermal Stability Screening results of CRL2^{FEM1B} and mutants.

(***A***-***F***) CRL2^{FEM1B} and mutants have similar melting temperatures (Tm), as shown by nanoDSF experiments (n=3).

(***G***-***L***) MBP-FBC and mutants have similar melting temperatures (Tm), as shown by nanoDSF experiments (n=3).

Appendix Table S1

Cryo-EM data collection, refinement and validation statistics

	CRL2^{FEM1B}	N8-CRL2^{FEM1B}-BEX2	N8-CRL2^{FEM1B}-FNIP1
PDB ID	8IJ1	8JE1	8JE2
EMDB ID	EMD-35461	EMD-36182	EMD-36183
**Data collection and processing**			
Magnification	130,000 ×	96,000 ×	105,000 ×
Voltage (kV)	300	300	300
Electron exposure ($e^-/Å^2$)	50	49.9	50
Defocus range (μm)	-1.5 to -2.0	-1.5 to -2.0	-1.5 to -2.0
Pixel size (Å)	0.92	0.86	0.83
Symmetry imposed	*C1*	*C1*	*C1*
Initial particle images (no.)	146,719	1,049,008	2,358,072
Final particle images (no.)	75,653	210,217	333,541
Map resolution (Å)	4.20	3.95	3.63
FSC threshold	0.143	0.143	0.143
Map resolution range (Å)	56.54-3.43	50.35-1.82	60.01-1.75
**Refinement**			
Initial model used (PDB code)	5N4W,6LBF	5N4W,6LBF	5N4W,7ROY
Model resolution (Å)	4.3	4.2	4.2
FSC threshold	0.5	0.5	0.5
Model resolution range (Å)	250-4.1	250-2.4	250-2.0
Map sharpening *B* factor ($Å^2$)	-56.74	-142.80	-132.85
Model composition			
Non-hydrogen atoms	25220	17508	9912
Protein residues	3172	2165	1237
Ligands	4	1	1
B factors ($Å^2$)			
Protein	157.75	138.62	109.07
Ligand	216.86	139.48	65.89
R.m.s. deviations			
Bond lengths (Å)	0.003	0.003	0.003
Bond angles (°)	0.596	0.715	0.666
Validation			
MolProbity score	1.74	1.97	1.87
Clashscore	10.73	11.74	12.62
Poor rotamers (%)	0.04	0.00	0.09
Ramachandran plot			
Favored (%)	96.88	94.32	96.23
Allowed (%)	3.09	5.68	3.77
Disallowed (%)	0.03	0	0
